# Notes

**Published:** 1898-03-26

**Authors:** 


### The Institutional Workshop.

### NOTES.

The building of the new Caird Hospital for Women, which includes a maternity department, at Dundee, is making rapid progress, and it is hoped the hospital will be ready for occupation in the autumn.

The erection of the new buildings of the Dental Hospital of London has been interfered with owing to a rise in price of building materials and difficulties arising in connection with public authorities. The committee calls attention to a decrease in annual sub scriptions.

The committee of the Dundee Infirmary are appealing for funds to enlarge their convalescent home at Barnhill. Although the home at present accommodates 45 patients pressure on this space is frequently so great as to make it necessary to retain convalescents in the infirmary while waiting for beds in the home.

The new Isolation Hospital at Hertford is fitted with a bacteriological laboratory. The Mayor of Hertford, at the opening ceremony, drew comparison between the old and the new use to which the hospital site has been put. The hospital is built on Gallows Hill, where formerly public executions took place. To-day, instead of so gruesome an association, people will look to the old hanging spot for kindness and healing.

Sir Alexander Mackenzie announced, on the occasion of the laying the foundation-stone of the New General Hospital at Calcutta, that the Government of India had consented to contribute half the amount necessary to support the hospital, the Bengal Government paying the other half. He also stated that the superintendent of the hospital will not be allowed to undertake private practice, and will be required to live in or near the hospital, the emoluments of the post being increased.

The extraordinary scandals which have been put into circulation, in regard to the Hospital for Sick Children at Bristol, and the practical breakdown of many of the charges brought against the institution, its medical staff, and its nursing, so soon as an attempt was made to put them into such form as to be capable of being investigated in accordance with ordinary rules of evidence, ought to teach hospitals how desirable it is that all charges and scandals should be at once investigated openly, independently, and in public view. The Commissioners appointed to inquire into the charges brought against the hospital by Dr. Christie, a former house surgeon, consisted of Mr. G. R. Askwith, a well-known member of the Western Circuit, who presided; the Hon. Arthur Elliot, barrister-at-law; Mr. Victor Williamson, C.M.G.; Dr. Church, of St. Bartholomew's Hospital; and Mr. Timothy Holmes, consulting surgeon to St. George's Hospital. With such a Commission, we may feel sure that the truth will be elicited, and that if errors of management should be discovered, as may well be the case, even though they fall far short of what has been charged against the hospital, the report when issued will have such weight that any evils which may be brought to light will at once be rectified; while if the charges should be declared to be frivolous, confidence in the hospital will be restored in a degree that would never have been the case had the investigation been less public and less complete.

